# Evaluation of tracer kinetic parameters in cervical cancer using dynamic contrast-enhanced MRI as biomarkers in terms of biological relevance, diagnostic performance and inter-center variability

**DOI:** 10.3389/fonc.2022.958219

**Published:** 2022-10-17

**Authors:** Xue Wang, Shujian Li, Xianhui Lin, Yi Lu, Chuanwan Mao, Zhijun Ye, Xuesheng Li, Tong-San Koh, Jie Liu, Jingjing Liu, Xiaoyue Ma, Jingliang Cheng, Gang Ning, Zhihan Yan, Zujun Hou

**Affiliations:** ^1^ Department of Radiology, The Second Affiliated Hospital and Yuying Children′s Hospital of Wenzhou Medical University, Wenzhou, China; ^2^ Department of Magnetic Resonance Imaging (MRI), The First Affiliated Hospital of Zhengzhou University, Zhengzhou, China; ^3^ Department of Pathology, The Second Affiliated Hospital and Yuying Children′s Hospital of Wenzhou Medical University, Wenzhou, China; ^4^ Department of Radiology, The Second Affiliated Hospital of Sichuan University, Chengdu, China; ^5^ Department of Oncologic Imaging, National Cancer Center, Singapore, Singapore; ^6^ The department of Jiangsu Key Laboratory of Medical Optics, Duke-National University of Singapore (NUS) Graduate Medical School, Singapore, Singapore; ^7^ Jiangsu Key Laboratory of Medical Optics, Suzhou Institute of Biomedical Engineering and Technology, Chinese Academy of Sciences, Suzhou, China

**Keywords:** imaging biomarker, dynamic contrast-enhanced imaging, reproducibility, multicenter study, cervix cancer

## Abstract

**Objectives:**

This study assessed the clinical value of parameters derived from dynamic contrast-enhanced (DCE) MRI with respect to correlation with angiogenesis and proliferation of cervical cancer, performance of diagnosis and reproducibility of DCE-MRI parameters across MRI scanners.

**Materials and Methods:**

A total of 113 patients with cervical carcinoma from two centers were included in this retrospective study. The DCE data were centralized and processed using five tracer kinetic models (TKMs) (Tofts, Ex-Tofts, ATH, SC, and DP), yielding the following parameters: volume transfer constant (Ktrans), extravascular extracellular volume (Ve), fractional volume of vascular space (Vp), blood flow (Fp), and permeability surface area product (PS). CD34 counts and Ki-67 PI (proliferation index) of cervical cancer and normal cervix tissue were obtained using immunohistochemical staining in Center 1.

**Results:**

CD34 count and Ki-67 PI in cervical cancer were significantly higher than in normal cervix tissue (p<0.05). Parameter Ve from each TKM was significantly smaller in cervical cancer tissue than in normal cervix tissue (p<0.05), indicating the higher proliferation of cervical cancer cells. Ve of each TKM attained the largest AUC to diagnose cervical cancer. The distributions of DCE parameters for both cervical cancer and normal cervix tissue were not significantly different between two centers (P>0.05).

**Conclusion:**

Parameter Ve was similar to the expression of Ki-67 in revealing the proliferation of tissue cells, attained good performance in diagnosis of cervical cancer, and demonstrated consistent findings on measured values across centers.

## Introduction

Cervical cancer is one of the top three most common cancers in women under 45 years old worldwide. Approximately 570,000 new cases and 311,000 deaths from cervical cancer occurred in 2018 (1, 2). Studies have proved that intra-tumoral microvessel density (MVD) are related strongly to tumor aggressiveness (such as invasive growth, lymphatic metastasis, and disease-free survival) (3–5). However, tumor MVD and its proliferation is generally obtained by immunohistochemical staining, which could be expressed by CD34 and ki-67 proliferation index (PI) after biopsy or operation. It would be desirable to identify biomarkers that can be used to assess tumor biology and to monitor the effects of treatment *in vivo*.

Dynamic contrast-enhanced magnetic resonance imaging (DCE-MRI) is a potential tool for characterizing tumor microcirculation. A variety of tracer kinetic models (TKMs) have been employed to diagnose various tumors and to assess the effects of anti-angiogenic and anti-vascular drugs in clinical trials (6–11). The Tofts model and the Extended Tofts (Ex-Tofts) model are frequently used for analysis of DCE-MRI data in clinical research or in clinical trials. A variety of two-compartment (the compartment of intravascular space and the extravascular extracellular space) models (2CXM) were proposed, which separately describe the intravascular transport using parameters blood (plasma) flow (Fp) and the exchange between the intravascular and the extravascular space using vessel permeability (PS), including the standard two-compartment model (SC), the adiabatic approximation to tissue homogeneity (ATH) and the distributed parameter model (DP) (12–15). Five DCE parameters (Ktrans, Fp, Vp, Ve, and PS) from above TKMs were obtained to assess tissue microcirculation. Interested readers can refer to Koh et al. (14) for a review on tracer kinetic modeling and the relevant clinical applications.

In spite of the advancement of tracer kinetic modeling and the promising results in various clinical studies, it remains challenging in developing robust imaging biomarkers, which require that imaging measurements sensitively capture the tissue biology of interest in a reliable and standardized fashion. A good quantitative biomarker should have three properties (16): biological relevance to the disease process under study, sensitivity to the disease process, and reliability (i.e., good reproducibility). Relevance and sensitivity could be established in single-center studies. Reproducibility of measurements might be good at single centers where the initial studies were carried out (to establish the sensitivity). However, at multiple centers, this will have to be established again.

Very few studies have been conducted to assess the kinetic parameters derived using DCE-MRI TKMs from the view of rigorous definition of biomarker, which has limited the widespread use of DCE parameters in clinical practice. This study attempted to (1) examine the relationship between DCE parameters and immunohistochemical indicators (CD34 and ki-67) in cervical cancer, (2) investigate the diagnostic performance of DCE parameters in differentiating cervical cancer and normal cervix tissue, and (3) evaluate the reproducibility of measured DCE parameters from various TKMs in cervical cancer patients using different scanners in a multicenter clinical setting.

## Materials and methods

### Subjects of study

This retrospective study was approved by the local ethics review boards in two institutions of this study. A total of 166 consecutive female patients, who were diagnosed with cervical carcinoma by histology and underwent MRI examination were reviewed in this study in the period of April 2016 to May 2021 in two centers. The inclusion criteria were: (1) patients diagnosed with cervical carcinoma by histology examination and (2) no history of chemoradiotherapy or surgery before MRI examination. Patients were excluded for the following reasons: (1) poor image quality of DCE-MRI such as significant motion artifacts or incomplete images (n=10), (2) patients with a history of targeted chemotherapy or radiation therapy before examination (n=16), (3) patients diagnosed with submucous myoma of uterus (n=5) and the endometrial carcinoma (n=4), and (4) no mass was identified for patients with stage Ia and Ib on DCE and other MRI sequences (n=18). Finally, 95 patients with cervix cancer and 18 cervical myoma were included in this retrospective study. ROIs (regions-of-interest) of normal tissue were obtained from cervical myoma and cervix cancer patients. ROIs for the tumor were obtained from cervix cancer patients.

### Imaging protocol

All MRI examinations were performed using two scanners: a 3T GE scanner (Discovery 750, GE Healthcare, Waukesha, WI, USA) from Center 1 and a 3T Siemens scanner (Skyra, Siemens AG, Erlangen, Germany) from Center 2.

T1-, T2-weighted and diffusion weighted images were acquired before intravenous administration of a gadolinium-based extracellular contrast agent (0.2 mmol/kg). The injection rate was 2~3 ml/s, with a dose of 0.1 mmol/kg body weight, followed by a 20 ml normal saline flush. DCE images were acquired in the axial plane under quiet respiration. After that, a routine late contrast-enhanced T1-weighted scan was acquired in the sagittal plane. Parameter settings of DCE imaging protocols were implemented based on the recommendation of Quantitative Imaging Biomarkers Alliance (QIBA) (17) but with improvement on temporal resolution according to the Nyquist-Shannon sampling theorem as detailed in **Table 1**.

**Table 1:** Parameters of DCE MRI acquisition protocol.

	Center 1	Center 2
**Vendor**	GE	Siemens
**Model**	Discovery 750	Skyra
**Field strength**	3.0T	3.0T
**Basic sequence**	LAVA	VIBE
**DCE protocol**		
**Pre-contrast FA**	4°, 8°, 11°	3°, 5°, 8°
**Post-contrast FA**	11°	8°
**TE (msec)**	1.2	1.2
**TR (msec)**	3.3	2.4
**FOV (mm2)**	360×360	380×285
**Matrix**	256×256	224×134
**Slice thickness (mm)**	5	5
**Number of slices**	6	20
**Pre-contrast phases**	10	10
**Post-contrast phases**	180	180
**Temporal resolution (sec)**	2.0	2.0

LAVA, liver acquisition with volume acquisition.

VIBE, volumetric interpolated breath-hold examination.

### Immunohistochemical assessment and histomorphometry of CD34 and Ki-67

Whether to perform the immunohistochemical analysis or not was according to the requirement of diagnosis in pathology or the treatment direction from clinician. Thus, immunohistochemical analysis with CD34 and Ki-67 may not be available for all the cervix cancer. In this study, the immunohistochemical analysis was performed in 14 cervix cancer and 12 cervix myoma. Some cervix masses were too large to accurately identify the normal cervix tissue in the visual field when reviewing the immunohistochemistry slides. Under 400 × magnification, we would exclude the CD34 count of the normal tissue if the number of CD34 of the normal tissue neighboring to the cervix mass was 0. Comparing with Ki-67, CD34 count of the normal tissue was evidently affected by the material limitation. Finally, the CD34 count and Ki-67 PI of cervix cancer mass were obtained from 14 cervix cancer patients. CD34 was obtained from 11 normal cervix tissue samples, including six cervix cancer and five cervix myoma. Ki-67 PI was obtained from 23 normal cervix tissue samples, including 13 cervix cancer and 10 cervix myoma.

The samples were fixed in 10% formalin and embedded in paraffin, according to standard procedures. A 3 μm thick sections were cut and mounted on glass slides. For each case, the routine hematoxylin and eosin staining, toluidine blue staining and immunohistochemical analysis with CD34 and Ki-67 were performed. Negative control was performed in immunohistochemical analysis. A gynecological pathologist with more than 6 years of experience reviewed the immunohistochemistry slides. The immunohistochemical analysis enabled calculation of two parameters:

(1) Microvessel density (MVD). CD34 of each tumor nuclei was labeled with CD34 monoclonal antibody (Maixin, Fuzhou, China). Single endothelial cell or clusters of endothelial cells positive for CD 34 was considered as a microvessel. The presence of blood cells or fibrin without any detectable endothelial cells is not sufficient to define a microvessel. Vessels with muscular walls were not counted. For each tumor, four hot spots (areas with the highest density of microvessels) were identified at low magnifications (×100). Subsequently, MVD was counted in each field (×400). The counts were expressed as the average of the four fields examined for each tumor.

(2) Ki-67 proliferation index (PI). Ki-67 of each tumor tissue was expressed as the percentage of tumor nuclei labeled with anti-MIB-1 monoclonal antibody (Maixin, Fuzhou, China). Under 400 × magnification, 1000 tumor cells were counted in 10 high-power visual fields at random. The Ki-67 PI was then defined as the number of positive cells/total cell count.

### Tracer kinetic models

DCE images were analyzed using a commercial software (MItalytics, FITPU Healthcare, Singapore). The following parameters were obtained: volume transfer constant (Ktrans, min−1) and extravascular extracellular volume (Ve, ml/100 ml) for Tofts; Ktrans, Ve, and fractional volume of vascular space (Vp, ml/100 ml) for Ex-Tofts; blood flow (Fp, ml/min/100 ml); permeability surface area product (PS, ml/min/100 ml), Vp, and Ve for ATH, SC, and DP. Details of the five tracer kinetic models (Tofts, Ex-Tofts, ATH, SC, and DP models) used in this study can be found in several review papers (13, 14, 18). For completeness, the operational equations of these models, which specify the dependence of tissue tracer concentration *C*_tiss_(t) (as a function of time t) on AIF and relevant physiological parameters were listed as follows:

Tofts model:


(1) $${\text{ C}}_{\text{tiss}}(t)=\text{ AIF }⊗{\text{K}}^{\text{trans}}\text{exp(}-\frac{{\text{K}}^{\text{trans}}}{{\text{v}}_{\text{e}}}t)$$

Ex-Tofts model:


(2) $${\text{ C}}_{\text{tiss}}(t)={\text{ AIF v}}_{\text{p}}+\text{AIF}⊗{\text{ K}}^{\text{trans}}\text{exp(}-\frac{{\text{K}}^{\text{trans}}}{{\text{v}}_{\text{e}}}t)$$

ATH model:


$${\text{C}}_{\text{tiss}}(t)=\text{AIF }⊗$$

(3) $${\text{F}}_{\text{p}}\left\{\begin{array}{l} \text{u(}t)-\text{u}\left(t-\frac{{\text{v}}_{\text{p}}}{{\text{F}}_{\text{p}}}\right)+\\ \text{u}\left(t-\frac{{\text{v}}_{\text{p}}}{{\text{F}}_{\text{p}}}\right)\{1-\text{exp}\left(-\frac{\text{PS}}{{\text{F}}_{\text{p}}}\right)\left[1+\int_{0}^{t-\frac{{\text{v}}_{\text{p}}}{{\text{F}}_{\text{p}}}}\text{exp}\left(-\frac{\text{PS}}{{\text{v}}_{\text{e}}}\text{τ}\right)\sqrt{\frac{\text{PS}}{{\text{v}}_{\text{e}}}\;\frac{\text{PS}}{{\text{F}}_{\text{p}}\;}\frac{1}{\text{τ}}}{\text{ I}}_{1}\left(2\sqrt{\frac{\text{PS}}{{\text{v}}_{\text{e}}}\;\frac{\text{PS}}{{\text{F}}_{\text{p}}}\text{τ}}\right)\text{dτ}\right]\} \end{array}\right\}$$

SC model:


(4a) $${\text{C}}_{\text{tiss}}(t)=\text{ AIF }⊗{\text{F}}_{\text{p}}[A\;\text{exp(α }t)+(1-A)\text{exp(β }t)],$$

where


(4b) $$\left(\begin{array}{c} \text{α}\\ \text{β} \end{array}\right)=\frac{1}{2}[-\left(\frac{\text{PS}}{{\text{v}}_{\text{p}}}+\frac{\text{PS}}{{\text{v}}_{\text{e}}}+\frac{{\text{F}}_{\text{p}}}{{\text{v}}_{\text{p}}}\right)\pm \sqrt{{\left(\frac{\text{PS}}{{\text{v}}_{\text{p}}}+\frac{\text{PS}}{{\text{v}}_{\text{e}}}+\frac{{\text{F}}_{\text{p}}}{{\text{v}}_{\text{p}}}\right)}^{2}-4\frac{\text{PS}}{{\text{v}}_{\text{e}}}\frac{{\text{F}}_{\text{p}}}{{\text{v}}_{\text{p}}}}],$$

(4c) $$\text{ A}=\frac{\text{α}+\frac{\text{PS}}{{\text{v}}_{\text{p}}}+\frac{\text{PS}}{{\text{v}}_{\text{e}}}}{\text{α}-\text{β}},\;$$

and

DP model:


$${\text{C}}_{\text{tiss}}(t)=\text{AIF}⊗$$

(5) $${\text{F}}_{\text{p}}\left\{\begin{array}{l} \text{u(}t)-\text{u}\left(t-\frac{{\text{v}}_{\text{p}}}{{\text{F}}_{\text{p}}}\right)+\\ \text{u}\left(t-\frac{{\text{v}}_{\text{p}}}{{\text{F}}_{\text{p}}}\right)\{1-\text{exp}\left(-\frac{\text{PS}}{{\text{F}}_{\text{p}}}\right)\left[1+\int_{0}^{t-\frac{{\text{v}}_{\text{p}}}{{\text{F}}_{\text{p}}}}\text{exp}\left(-\frac{\text{PS}}{{\text{v}}_{\text{e}}}\text{τ}\right)\sqrt{\frac{\text{PS}}{{\text{v}}_{\text{e}}}\;\frac{\text{PS}}{{\text{F}}_{\text{p}}\;}\frac{1}{\text{τ}}}{\text{ I}}_{1}\left(2\sqrt{\frac{\text{PS}}{{\text{v}}_{\text{e}}}\;\frac{\text{PS}}{{\text{F}}_{\text{p}}}\text{τ}}\right)\text{dτ}\right]\} \end{array}\right\}$$

### Image post-processing

**Figure 1:**
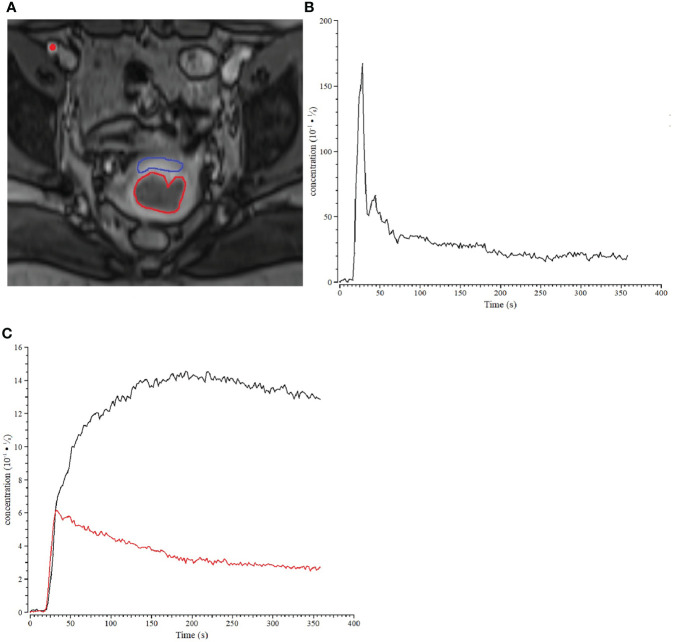
Example of a patient with stage IIb cervix cancer. **(A)** ROIs for cervix cancer (bred) and normal cervix tissue (blue) are shown for one slice of the DCE-MRI dataset, and the location within the iliac artery where the AIF was sampled was marked with a red dot. **(B)** Sample of AIF for the cervix cancer patient. **(C)** The concentration-time curve of cervix cancer (red) ROI and the normal tissue (black).

For each patient, ROIs for the tumor and the normal tissue were manually delineated on the central slices of DCE images (to avoid possible effects of inflow and inhomogeneity near boundaries) by a radiologist with more than 10 years of experience in gynecological radiology. Routine T1-weighted, T2-weighted, and DW images were referenced to currently delineate ROIs. The size of ROI was no less than 10 voxels to ensure robustness of measurement. The normal ROIs were selected in the normal cervix tissue away from the lesions. The areas of necrotic, cystic, and hemorrhages were avoided when drawing the lesion ROIs. All ROIs were confirmed by a senior radiologist, and disagreements were resolved with consensus-based discussion. The arterial input function (AIF) was sampled from a voxel that clearly resided within the external iliac artery on one of the central slices. Desirable features for AIF selection included an early bolus arrival time, high peak value and signal-to-noise ratio. The sampled AIF and concentration-time curve of cervix cancer ROI and the normal tissue are showed in **Figure 1**. The concentration of normal tissue is higher than the tumor in each phase. In later phase, the enhanced pattern of normal tissue is “persist”, and the cancer is “wash out”.

### Statistical analysis

The median parameter value of voxels in tumor ROIs on multiple slices for each patient was taken as a representative statistic of the parameter. Kolmogorov-Smirnov test was conducted to analyze the normality of CD34 counts, Ki-67 PI in Center 1, and DCE parameters in two centers. Independent sample t-test was used to compare the differences of CD34 counts between cervix cancer and the normal tissue in Center 1. Mann–Whitney U test was used to compare the differences of Ki-67 PI between cervical cancer and normal cervix tissue in Center 1. Pearson correlation coefficient r was used to explore possible relationship between immunohistochemical indicators (CD34 and Ki-67) and DCE kinetic parameters from the five models (Ex-Tofts, Tofts, ATH, SC, and DP) of cervix lesion and normal tissue in Center 1. A strong correlation was assumed for 0.8 < r ≤ 1, a moderate correlation for 0.5 < r ≤ 0.8, a weak correlation for 0.3 < r ≤ 0.5, and no correlation for r ≤ 0.3 (19). Receiver operating characteristic (ROC) analysis was performed to examine the ability of each parameter of the two centers in discriminating cervix tumor and normal tissue, and the discriminating power of each parameter was quantified using the area under ROC curve (AUC). Interpretation of AUC values is application-dependent, and in general, it is appropriate that values ≥0.9 would be “excellent”, ≥0.8 “good”, ≥0.7 “fair”, and<0.7 “poor” (20). Mann–Whitney U test was used to compare the distribution differences of various parameters in two centers. P<0.05 indicated statistical significance. Analyses were performed using SPSS Statistics (version 21.0, IBM Corp., Armonk, NY, USA).

## Results

### Study population

**Figure 2:**
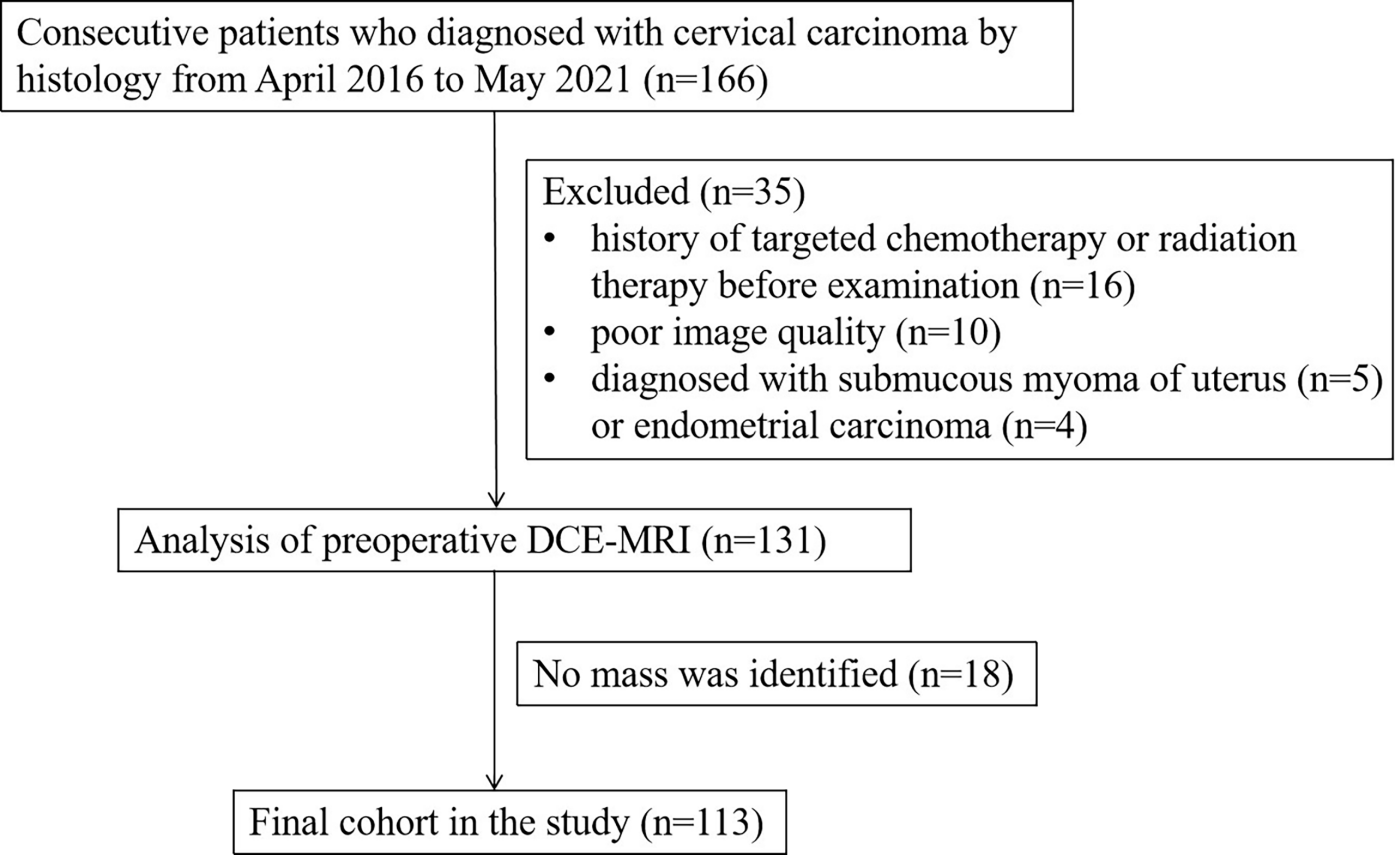
Flowchart of patient population.

Of the 166 cases, 113 cases met the criteria of inclusion and formed the final study cohort with age mean and range of 56 years (37–75 years) and 48.5 years (31–72 years) in Center 1 and Center 2, respectively (**Figure 2**). Characteristics for cervix cancer patients were summarized in **Table 2**.

**Table 2:** Cervix cancer patient characteristics.

Characteristics	Center 1(n=47)	Center 2(n=48)
Age average (range)	56(37–75)	48.5(31–72)
Histologic type		
Squamous cell carcinoma (SCC)	45	39
Adenocarcinoma	2	6
Adenosquamous carcinoma	0	3
Grade		
G1	13	3
G2	17	4
G3	2	34
Not graded	15	7
Clinic stage		
Ia	6	3
Ib	17	33
IIa	12	7
IIb	7	3
IIIa	3	2
IIIc	1	0
IVa	1	0
IVb	0	0
Treatment before MR examination		
Chemoradiotherapy	0	0
Surgery	0	0
No Treatment	47	48

### Cervical cancer microenvironment characterization in center 1

**Figure 3:**
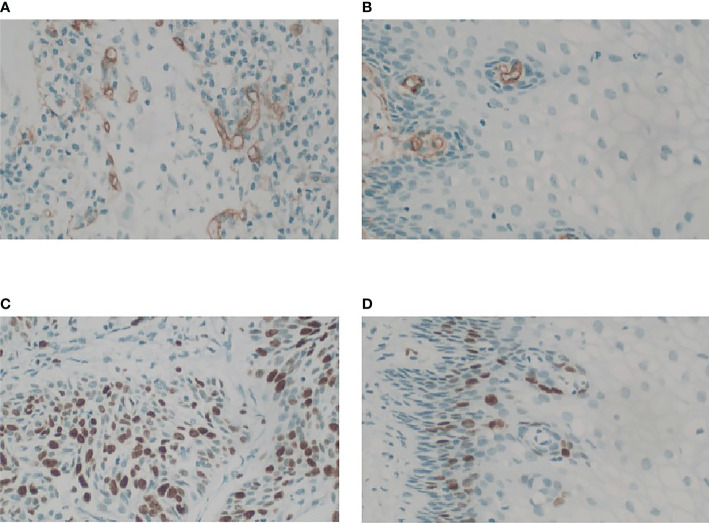
Microphotograph showing MVD and Ki-67 PI in cervix cancer mass and the normal tissue for stage IIb cervix cancer in Center 1 (GE 750). **(A)** Magnification (×40) of one of the hot spots in cervix cancer reveals a high histological microvessel density. **(B)** Magnification (×40) of one of the hot spots in normal cervix tissue reveals a low histological microvessel density. **(C)** Immunostain of a cervix cancer for Ki67 showing labelling of roughly 70% of nuclei, 40×. **(D)** Immunostain of the normal cervix tissue for Ki67 showing positive expression is located in the base, 40×.

CD34 counts in cervical cancer (20.35 ± 5.82) were significantly higher than in normal cervix tissue (5.98 ± 2.77) (P<0.05). Ki-67 PI in cervical cancer (65% ± 29%) was significantly higher than in normal cervix tissue (1%) (P<0.05) (**Figure 3**). Pearson correlation between immunohistochemical indicators (CD34 and Ki-67) and DCE kinetic parameters from the five models (Ex-Tofts, Tofts, ATH, SC, and DP) of cervix lesion and normal tissue in Center 1 were showed in **Table 3**. For Ex-Tofts and Tofts models, parameter Ktrans was negatively correlated with Ki-67 PI (r>0.5, P<0.05) for cervical cancer, and weak or little correlation was observed between parameters Vp or Ve and Ki-67 PI (r<0.4, *P >*0.05). For 2CXMs (ATH, SC, and DP), Vp was negatively correlated with Ki-67 PI (r>0.6, P<0.05) for cervical cancer. Weak or little correlation was observed in either cervical cancer or normal cervix tissue between Ve and Ki-67 PI (r<0.3, P>0.05). Inconsistent correlation across 2CXMs in cervical cancer was shown between PS and Ki-67 PI (r=-0.489, P=0.076 for ATH; r=0.218, P=0.454 for SC; and r=-0.143, P=0.627 for DP, respectively). Moderately, negative correlation was noted on Fp from SC and DP with Ki-67 PI in cervical cancer (r=-0.520, P=0.057 for SC; r=-0.537, P=0.047 for DP).

**Table 3:** Results of Pearson correlation immunohistochemical indicators (CD34 and Ki-67) and DCE kinetic parameters (Ktrans, Fp,Vp, Ve, PS) from Tofts, Ex-Tofts, ATH, SC, and DP models of cervix lesion and normal tissue in Center 1, withcorrelation coefficients and p-values in the bracket.

	Ktrans (min^−1^)	Fp (ml/min/100 ml)	Vp (ml/100 ml)	Ve (ml/100 ml)	PS (ml/min/100 ml)
Tofts model					
Cervix lesion					
CD34	-0.024 **(**0.936**)**	–	–	0.09 (0.759)	–
Ki-67	-0.550 (0.041)	–	–	-0.216 (0.459)	–
Normal tissue					
CD34	-0.369 (0.264)	–	–	-0.473 (0.142)	–
Ex-Tofts model					
Cervix lesion					
CD34	-0.028 **(**0.924**)**	–	0.353 (0.215)	0.07 (0.813)	–
Ki-67	-0.554 (0.04)	–	-0.321 (0.263)	-0.252 (0.384)	–
Normal tissue					
CD34	-0.404 (0.218)	–	0.40 (0.223)	-0.491 (0.125)	–
ATH model					
Cervix lesion					
CD34	–	-0.046 **(**0.877**)**	0.068 (0.817)	0.065 (0.824)	-0.209 (0.473)
Ki-67	–	-0.057 (0.847)	-0.661 (0.01)	-0.198 (0.430)	-0.489 (0.076)
Normal tissue					
CD34	–	-0.622 (0.041)	0.357 (0.282)	-0.456 (0.158)	-0.493 (0.123)
CC model					
Cervix lesion					
CD34	–	0.08 **(**0.786**)**	-0.125 (0.669)	0.071 (0.808)	-0.389 (0.170)
Ki-67	–	-0.520 (0.057)	-0.747 (0.002)	-0.065 (0.824)	0.218 (0.454)
Normal tissue					
CD34	–	-0.146 (0.668)	-0.243 (0.471)	-0.245 (0.469)	-0.340 (0.307)
DP model					
Cervix lesion					
CD34	–	0.023 **(**0.938**)**	0.080 (0.787)	0.033 (0.911)	-0.329 (0.250)
Ki-67	–	-0.537 (0.047)	-0.673 (0.008)	-0.268 (0.355)	-0.143 (0.627)
Normal tissue					
CD34	–	-0.055 (0.872)	-0.198 (0.559)	-0.113 (0.742)	-0.41 (0.164)

Correlations between DCE-MRI parameters and CD34 in cervical cancer or normal cervix tissue were largely weak or not correlated, except for Fp from ATH in normal cervix tissue (r=-0.622, P<0.05).

### Diagnostic performance of DCE-MRI parameters in differentiating cervical cancer from normal cervix tissue

For Center 1, 47 ROIs for cervical cancer were obtained. A total of 63 ROIs for normal tissues were obtained from 16 cervix myoma and 47 cervical cancer patients. For Center 2, 48 ROIs for cervical cancer were obtained. A total of 50 ROIs for normal tissues were obtained from two cervix myoma and 48 cervical cancer patients.

**Figure 4:**
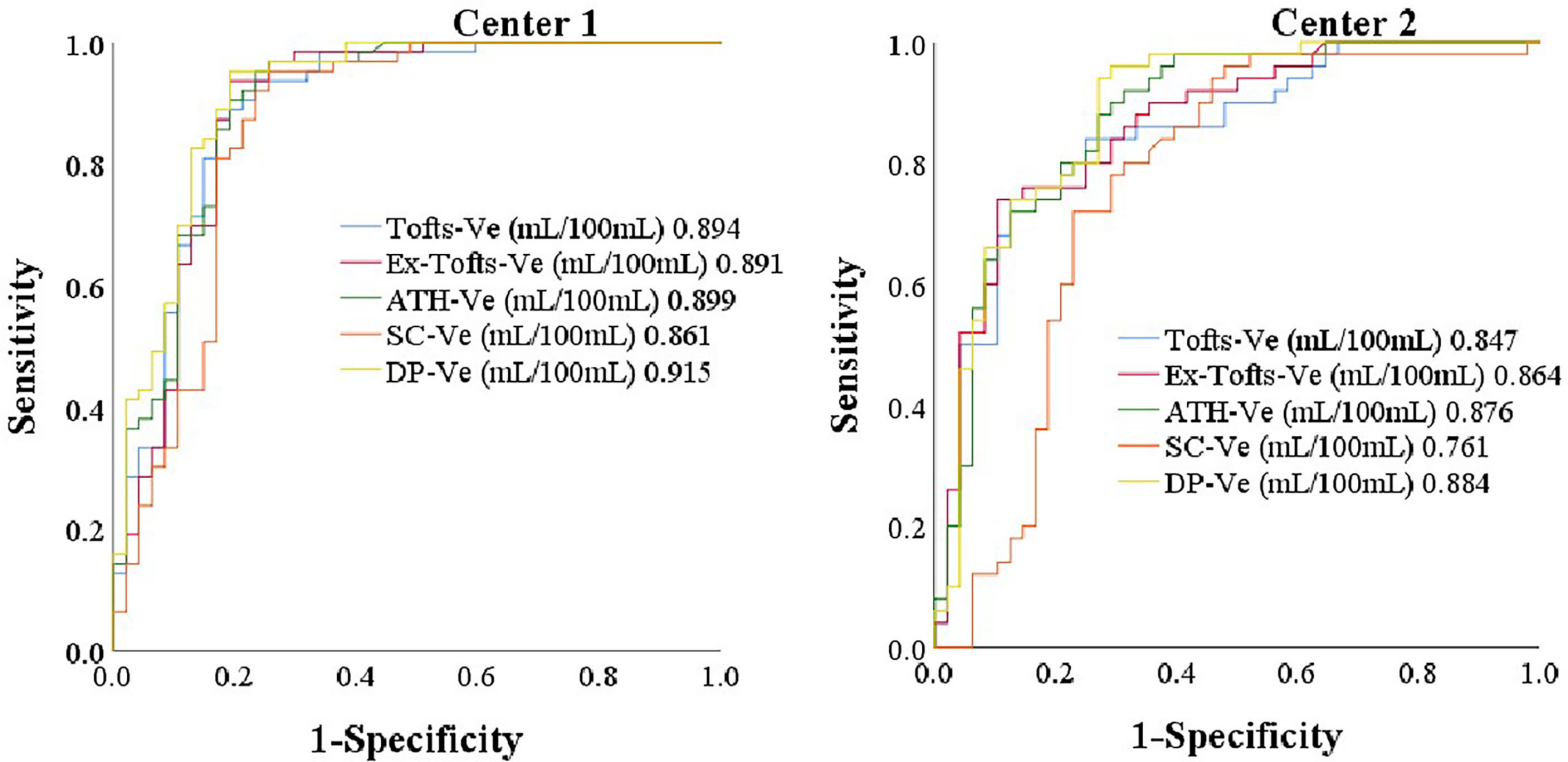
The ROC of parameter Ve from the five models (Ex-Tofts, Tofts, ATH, SC, and DP) in Center 1 and Center 2, respectively.

**Figure 5:**
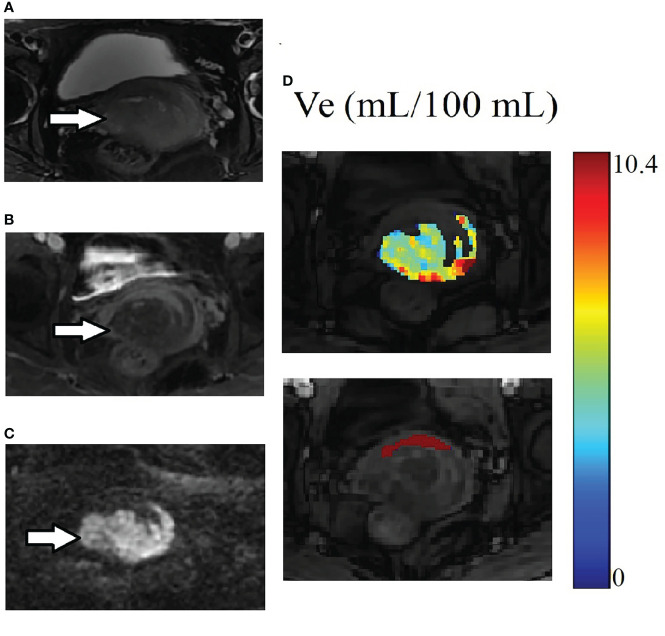
Example of MRI scans for the same patient in **Figure 3**. **(A)** Cervix cancer exhibits slightly high signal intensity on axial T2-weighted image. **(B)** The degree of enhancement for cervix cancer is lower than the normal cervix tissue on delayed contrast imaging. **(C)** Cervix cancer exhibits high signal intensity on DW image. **(D)** The parameter Ve maps generated using the ATH model for cancer and the normal tissue ROIs. The upper and lower images are for cancer and the normal tissue ROIs respectively. Parameter Ve value of cervix cancer is significantly smaller than that of normal cervix tissue.

AUC values of DCE kinetic parameters derived by five models (Ex-Tofts, Tofts, ATH, SC, and DP) in differentiating cervical carcinoma tissue from normal cervix tissue in two centers were listed in **Table 4**, where Ve attained the largest AUC in each TKM. **Figure 4** showed the ROC of parameter Ve from the five models (Ex-Tofts, Tofts, ATH, SC, and DP) in Center 1 and Center 2, respectively. At least one parameter in each TKM attained good performance (AUC value > 0.8) to diagnose cervical cancer in both centers, except for parameter of SC model in Center 2 (the highest AUC value=0.761). **Figure 5** showed the parameter (Ve) maps generated using the ATH model for cervix cancer and the normal tissue.

**Table 4:** AUC values of DCE kinetic parameters derived by various models in differentiating cervical carcinoma tissue from normal cervix tissue in the three centers.

DCE kinetic parameters	Center 1	Center 2
Tofts-Ktrans	0.503	0.517
Tofts-Ve (ml/100 ml)	0.894	0.847
Ex-Tofts–Ktrans	0.571	0.504
Ex-Tofts–Vp (ml/100 ml)	0.669	0.544
Ex-Tofts–Ve (ml/100 m)	0.891	0.864
ATH-Fp (ml/min/100 ml)	0.783	0.658
ATH-Vp (ml/100 ml)	0.693	0.508
ATH-Ve (ml/100 ml)	0.899	0.876
ATH-PS (ml/min/100 ml)	0.627	0.555
SC-Fp (ml/min/100 ml)	0.506	0.538
SC-Vp (ml/100 ml)	0.55	0.614
SC-Ve (ml/100 ml)	0.861	0.761
SC-PS (ml/min/100 ml)	0.782	0.668
DP-Fp (ml/min/100 ml)	0.505	0.504
DP-Vp (ml/100 ml)	0.504	0.612
DP-Ve (ml/100 ml)	0.915	0.884
DP-PS (ml/min/100 ml)	0.681	0.610

### Reproducibility of DCE-MRI parameters across centers

The distribution differences among various parameters of cervical cancer and normal tissue in different centers were assessed by Man–Whitney U test, and shown in **Table 5** for the five models (Tofts, Ex-Tofts, ATH, SC, and DP). The distribution of parameters Ktrans, Ve from Tofts for both cervix cancer and normal tissue, showed similar in Center 1 and Center 2 (P>0.05). The distribution of parameters Fp, Vp, Ve, PS from ATH for cervix cancer showed similar in Center 1 and Center 2 (P>0.05). The distribution of at least three parameters from SC and DP models for cervix cancer showed similar between the two centers (P>0.05).

**Table 5:** Measured values (median and inter-quantile range in the bracket) of DCE kinetic parameters (Ktrans, Fp,Vp, Ve, PS) derived from (Tofts, Ex-Tofts, ATH, SC, and DP models) in cervical cancer and normal cervix tissue, and the corresponding p-values of Mann–Whitney U test.

	Ktrans (min^−1^)	Fp (ml/min/100 ml)	Vp (ml/100 ml)	Ve (ml/100 ml)	PS (ml/min/100 ml)
Tofts model					
Cervix lesion					
Center 1	0.09 (0.08,0.13)	–	–	13.91 (11.32,17.30)	–
Center 2	0.13 (0.08,0.19)	–	–	13.97 (10.07,17.61)	–
*Z* value	-1.858	–	–	-0.074	–
*P *value	0.063	–	–	0.941	–
Normal tissue		–	–		–
Center 1	0.11 (0.07,0.14)	–	–	30.74 (22.40,42.62)	–
Center 2	0.12 (0.08,0.16)	–	–	28.46 (19.29,39.73)	–
*Z* value	-1.595			-1.350	
*P *value	0.111	–	–	0.177	–
Ex-Tofts model					
Cervix lesion					
Center 1	0.07 (0.05,0.10)	–	1.71 (1.21,2.34)	12.65 (10.05,17.30)	–
Center 2	0.10 (0.06,0.16)	–	1.45 (1.02,2.20)	14.04 (11.14,17.78)	–
*Z* value	-2.066	–	-1.042	-1.589	–
*P *value	0.039	–	0.297	0.112	–
Normal tissue		–			–
Center 1	0.09 (0.05,0.12)	–	1.08 (0.27,2.06)	33.75 (24.63,45.38)	–
Center 2	0.10 (0.07,0.13)	–	1.72 (0.93,2.80)	29.77 (19.93,41.44)	–
*Z* value	-1.333		-1.827	-2.220	
*P *value	0.182	–	0.068	0.026	–
ATH model					
Cervix lesion					
Center 1		21.88 (17.82,35.43)	2.43 (1.26,4.03)	11.16 (9.17,15.58)	7.10 (4.78,9.54)
Center 2		26.36 (18.03,36.74)	2.64 (1.64,3.97)	12.32 (8.62,17.35)	9.44 (5.61,13.21)
*Z* value		-.897	-.707	-.175	-1.924
*P *value		0.370	0.479	0.861	0.054
Normal tissue					
Center 1		39.25 (31.65,51.61)	1.00 (0.39,2.56)	33.89 (23.94,45.93)	9.57 (5.70,13.34)
Center 2		35.37 (24.98,46.73)	2.72 (1.43,6.29)	27.52 (18.14,38.91)	9.74 (6.43,14.76)
*Z* value		-1.908	-3.645	-2.197	-0.853
*P *value		*P*=0.056	*P<* 0.001	0.028	0.394
SC model					
Cervix lesion					
Center 1		15.26 (12.61,21.78)	6.12 (4.00,8.62)	8.74 (6.36,14.14)	4.76 (2.73,8.29)
Center 2		19.06 (15.40,30.82)	6.39 (5.01,8.27)	10.88 (6.68,19.83)	5.52 (3.13,10.82)
*Z* value		-2.791	-0.197	-0.867	-0.878
*P *value		0.005	0.844	0.386	0.380
Normal tissue					
Center 1		16.65 (12.15,20.51)	5.70 (2.99,9.91)	26.39 (19.25,39.25)	13.47 (6.61,22.14)
Center 2		22.14 (15.96,34.69)	8.49 (5.42,12.58)	31.11 (18.25,46.92)	9.39 (6.37,16.73)
*Z* value		-3.512	-2.321	-0.425	-1.734
*P *value		*P<* 0.001	0.02	0.671	0.083
DP model					
Cervix lesion					
Center 1		13.83 (11.64,17.00)	3.04 (2.08,5.37)	10.56 (8.63,14.83)	6.81 (4.92,9.33)
Center 2		14.81 (0.05,0.10)	4.04 (3.43,6.14)	10.48 (7.73,15.39)	8.31 (5.68,12.22)
*Z* value		-1.455	-2.099	-0.462	-1.012
*P *value		0.146	0.036	0.644	0.311
Normal tissue					
Center 1		13.93 (0.08,0.13)	3.77 (1.80,6.03)	29.82 (23.59,39.35)	11.63 (6.21,17.87)
Center 2		16.22 (11.27,22.54)	5.58 (4.09,8.80)	24.52 (17.35,35.95)	11.46 (7.30,14.95)
*Z* value		-2.017	-3.226	-2.208	-0.801
*P *value		.044	0.001	.027	.423

## Discussion

In this study, we investigated the potential of DCE-MRI parameters as biomarkers with respect to correlation with angiogenesis and proliferation of cervical cancer, performance of differential diagnosis, and reproducibility of DCM-MRI parameters across MRI scanners in different centers. It was turned out that Ktrans of Tofts or Ex-Tofts, Vp of three 2CXMs, Fp of ATH, and DP, showed moderately negative correlation with Ki-67 in cervical cancer tissue, and Fp of ATH showed moderately negative correlation with CD34 in normal cervix tissue. Ve of each TKM attained the largest AUC. No significant differences were observed on the distributions of most DCE-MRI parameters in either cervical cancer or normal cervix tissue between Center 1 and Center 2, indicating certain degree of reproducibility between these two scanners.

As a transmembrane glycoprotein expressed in capillary endothelial cells, CD34 is a useful angiogenesis marker reflecting the grade of microvascular modeling in cervical cancer (21). This study found that the expression of CD34 in cervical cancer tissue was significantly higher than that in normal cervix tissue, indicating that there is an increased neovascularization in cervical cancer tissue. In DCE tracer kinetic modeling, Vp reflects the fractional volume of intravascular space, with measurement corresponding to tissue MVD. However, measured Vp values in TKMs were smaller in cervical cancer than in normal cervix tissue, and showed little correlation with the expression of CD34. Hauge et al. (22) investigated the potential of DCE-MRI to assess MVD using patient-derived cervical cancer xenografts and found that none of the DCE-MRI parameters was related to MVD. The discrepancy could be explained as follows. As pointed out by Hylton (23), MVD, as measured using immunohistopathological method, gives a partial picture of the tissue microvasculature, but does not reflect the functional property of microvasculature, including permeability, which contributes to the DCE-MRI measurement. In addition, MVD is also a heterogeneous property of tumors. MVD measurement methods are limited by histopathologic sampling and are generally hotspot values, which are, by definition, localized. Accurate correlation necessitates precise comparison of anatomical MRI maps with whole-mount histological tumor specimens rather than with biopsy specimens that may only represent a small sample of the tumor, so that comparably sized and geometrically oriented regions of interest can be compared (24).

Instead of assessing direct association between global DCE-MRI parameter and local histopathologic sampling data, a more reasonable way could be relative comparison with self-reference. In the context of current study, study object is cervical cancer tissue and reference object is normal cervix tissue. Either DCE-MRI parameter or histopathologic indicator independently forms two sampled datasets in cervical cancer tissue and reference tissue, from which sample statistic can be estimated and inference between cervical cancer and reference tissue can be conducted. With this in mind, we can proceed to the interpretation of parameter Ve from the view point of biomarker.

Ve measures the fractional volume of extravascular extracellular space, which is inversely related to cellular density and could be linked to cell proliferation. Sustained proliferation of cells is one of the most important characteristics of cancer (25). Ki67, a nuclear antigen expressed in the nucleus of cells in active proliferation, is considered a valid nuclear marker of cell proliferation. Studies revealed that Ki67 is highly expressed in proliferative cells in many kinds of cancers, but rarely in normal cells (26, 27). As shown in the results, the expression of Ki-67 in cervical cancer tissue (65% ± 29%) was significantly higher than in normal cervix tissue (1%), indicating the higher proliferation of cervical cancer cells. On the other hand, measured values of Ve of each TKM were significantly smaller in cervical cancer tissue than in normal cervix tissue (**Table 5**), suggesting the higher density of cervical cancer cells, which was in accordance with the findings from Ki-67 expression. In addition, Ve almost attained the highest diagnostic performance in differentiating cervical cancer from normal cervix tissue by all TKMs, and the measured Ve values showed certain degree of reproducibility between Center 1 and Center 2.

The study had the following limitations. The experiment of immunohistopathologic staining was limited to a subset of cases in one center for patients for whom it was requested by the physician, which may have introduced bias. It would be desirable to conduct the experiment in a larger dataset across different centers. The size of study cohort in each center was relatively small, and the differences of sample demographics and tumor grade distribution could be prone to sources of variations between the two centers. Next step, we will continue to explore the issue of parameter reproducibility in a larger cohort with the same grade cervix cancer among centers.

In conclusion, this study assessed the potential of DCE-MRI kinetic parameters as biomarkers in cervical cancer and found that, in each tracer kinetic model, parameter Ve was similar to the expression of Ki-67 in reflecting tissue cell proliferation, attained good performance in differential diagnosis of cervical cancer and normal cervix tissue, and demonstrated results on measured values across centers without significant difference between distributions. From this point of view, Ve measurements derived from primary tracer kinetic models were equally applicable as potential imaging biomarkers in cervical cancer diagnosis.

## Data availability statement

The raw data supporting the conclusions of this article will be made available by the authors, without undue reservation.

## Ethics statement

This study was reviewed and approved by Ethics Committee of the Second Affiliated Hospital and Yuying Children′s Hospital of Wenzhou Medical University. The ethics committee waived the requirement of written informed consent for participation.

## Author contributions

XW and SL co-first authors because they did data collection, compilation and postprocessing, literature study and manuscript drafting, and neither of them is a trainee. ZY and ZH were the senior author and corresponding author respectively, because they contributed the conception of the work, were responsible for the execution of the project in the respective institution, facilitated the progress of this multicenter study, revised the manuscript and approved the final manuscript for submission. Material preparation, data collection and analysis were performed by XiL, YL, ZJY, XuL, T-SK, JL, JJL, XM, JC, GN. All authors contributed to the article and approved the submitted version.

## Funding

This study has received funding from Wenzhou Science and Technology Buteau in China (No.Y20220070) and Zhejiang Provincial Medical and Health Project (No.2023RC212).

## Acknowledgments

The authors were grateful to Mr Liuyang Chen of Fitpu Healthcare for his assistance in software customization during data processing.

## Conflict of interest

The authors declare that the research was conducted in the absence of any commercial or financial relationships that could be construed as a potential conflict of interest.

## Publisher’s note

All claims expressed in this article are solely those of the authors and do not necessarily represent those of their affiliated organizations, or those of the publisher, the editors and the reviewers. Any product that may be evaluated in this article, or claim that may be made by its manufacturer, is not guaranteed or endorsed by the publisher.
